# Potential Effects of Poloxamer 188 on Rat Isolated Brain Mitochondria after Oxidative Stress In Vivo and In Vitro

**DOI:** 10.3390/brainsci11010122

**Published:** 2021-01-18

**Authors:** Johannes A. Pille, Matthias L. Riess

**Affiliations:** 1 Department of Anesthesiology, Vanderbilt University Medical Center, Nashville, TN 37232, USA; johannespille@aol.de; 2 Department of Anesthesiology, University Medicine Greifswald, 17475 Greifswald, Germany; 3 Department of Pharmacology, Vanderbilt University, Nashville, TN 37232, USA; 4 Anesthesiology, TVHS VA Medical Center, Nashville, TN 37212, USA

**Keywords:** asphyxia, ATP synthesis, calcium retention capacity, cardiac arrest, copolymer, cerebral, H_2_O_2_, ischemia reperfusion injury, oxygen consumption, P188

## Abstract

Outcome after cerebral ischemia is often dismal. Reperfusion adds significantly to the ischemic injury itself. Therefore, new strategies targeting ischemia/reperfusion (I/R) injury are critically needed. Poloxamer (P)188, an amphiphilic triblock copolymer, is a highly promising pharmacological therapeutic as its capability to insert into injured cell membranes has been reported to protect against I/R injury in various models. Although mitochondrial function particularly profits from P188 treatment after I/R, it remains unclear if this beneficial effect occurs directly or indirectly. Here, rat isolated brain mitochondria underwent oxidative stress in vivo by asphyxial cardiac arrest or in vitro by the addition of hydrogen peroxide (H_2_O_2_) after isolation. Mitochondrial function was assessed by adenosine triphosphate synthesis, oxygen consumption, and calcium retention capacity. Both asphyxia and H_2_O_2_ exposure significantly impaired mitochondrial function. P188 did not preserve mitochondrial function after either injury mechanism. Further research is indicated.

## 1. Introduction

Cerebral ischemia continues to dramatically affect many patients each year, even though newer and better therapies are constantly being developed [1,2,3,4]. Reperfusion, i.e., the return of blood flow after ischemia—while absolutely necessary—has been determined as a strong contributing factor to cell death, though, leading to the term I/R injury [5,6].

Interestingly, mitochondria seem to play a crucial role in I/R injury, making them an attractive target for potential therapies [5,7,8]. The important role of mitochondria in I/R injury is the subject of increasing research as mitochondria are negatively affected during I/R but also contribute to deteriorating intra- and extracellular reactions [5,7].

The lack of oxygen (O_2_) causes inhibition of the respiratory chain and, consequently, of the adenosine triphosphate (ATP) synthase. To sustain the mitochondrial membrane potential (Δψ_m_), the ATP synthase will even start running in reverse mode through hydrolyzation of ATP, further decreasing cellular ATP levels [5]. Without oxidative phosphorylation, the tricarboxylic acid cycle and β-oxidation will be impeded as well, and toxic fatty acids with pro-inflammatory properties accumulate [5].

Inhibition of the respiratory chain also causes the production of reactive oxygen species (ROS) in multiple ways [5,7]. The cell’s antioxidant system tries to buffer these but becomes overwhelmed quickly [5].

The formation and opening of the mitochondrial permeability transition pore (mPTP) are additional critical events during I/R injury [5]. Mitochondrial permeability transition (MPT) is triggered by many circumstances during I/R [5]. It causes a complete collapse of the Δψ_m_ as protons (H^+^) easily pass through the mPTP into the matrix [5,8]. This transition of H^+^ pulls water following the osmotic gradient into the mitochondrial matrix, which causes mitochondrial swelling and possibly even rupture [5,8].

Although the triblock copolymer Poloxamer (P)188, an amphiphilic triblock copolymer, has been shown to protect different cell types, likely based on its cell-membrane-sealing effect [9,10,11,12,13,14,15,16,17,18,19], the frequently observed beneficial effect on mitochondrial function remains intriguing [9,18]. It is possible that the protection of cell membranes with subsequent cellular recovery generally leads to better preserved mitochondrial function as an indirect, downstream consequence. However, mitochondria are also encapsulated by biolipid membranes, not only by one but two [20], which can be severely damaged during I/R injury as well.

Therefore, we hypothesized that P188 has a sealing effect on mitochondrial membranes and subsequently preserves mitochondrial function against I/R injury directly. Thus, we aimed at:

(a) Developing an in vitro I/R injury model through exposure of rat isolated forebrain mitochondria to the ROS hydrogen peroxide (H_2_O_2_) after isolation;
(b) Developing an in vivo I/R injury model through asphyxial cardiac arrest (CA) in rats before isolation of forebrain mitochondria;
(c) Assessing potential positive effects of P188 on isolated, injured mitochondria by measuring ATP synthesis, O_2_ consumption, and calcium retention capacity (CRC).

## 2. Materials and Methods

All drugs were purchased from Sigma (St. Louis, MO, USA) unless otherwise indicated.

### 2.1. Animals

The investigation conformed to the Guide for the Care and Use of Laboratory Animals (Institute for Laboratory Animal Research, National Academy of Sciences, 8th edition, 2011) and was approved by the Institutional Animal Care and Use Committee (M1600012, 29 January 2018; M1700168, 28 October 2017; M1800029, 16 April 2018). All animals used for the experiments were male Sprague–Dawley rats (350–450 g).

#### 2.1.1. Animal Preparation for Euthanasia

Twenty-two rats were anesthetized with intraperitoneal administration of 100 mg×kg^−1^ ketamine (KetaVed^®^, Vedco Inc., Saint Joseph, MO, USA). If not enough after 15 min, a supplementary dose of 5 mg×kg^−1^ was administered every 10 min until the animal did not show any reaction to tail and toe pinch. Five min before euthanasia by decapitation, 3000 U kg^−1^ heparin (West-Ward Pharmaceuticals, Eatontown, NJ, USA) was administered.

#### 2.1.2. Animal Preparation for Asphyxial CA

To prepare for asphyxial CA, 6 rats were anesthetized with intraperitoneal administration of 50 mg×kg^−1^ pentobarbital (Diamondback Drugs, Scottsdale, AZ, USA). Supplementary doses with 10 mg×kg^−1^ were given if needed until the animal was nonreactive to pain stimuli. Following a protocol modified from Lamoreux et al. [21], the animals were intubated; vascular access was achieved via cannulation of the tail vein and surgical cutdown of femoral vein and artery. Electrocardiogram (ECG) via subcutaneous ECG needles, rectal temperature, arterial blood pressure, and venous waveforms were recorded using Powerlab and LabChart (AD Instruments, Colorado Springs, CO, USA).

Rats were ventilated (Small Animal Ventilator, Model 683, Harvard Apparatus Inc., Holliston, MA, USA) with a tidal volume of 0.6 mL×kg^−1^, FiO_2_ 0.25, positive end-expiratory pressure of 5 cmH_2_O, and a respiratory rate adjusted to achieve an endtidal CO_2_ of 40 ± 5 mmHg, measured by an infrared CO_2_ Sensor (Capnogard, Novametrix, Wallingford, CT, USA). Rats were then randomized into CA vs. a sham procedure (see below).

#### 2.1.3. Brain Extraction

The anesthetized animal was decapitated with a guillotine. The skull was cut open with scissors, starting from the occipital bone and continuing to splice up the suture between the parietal bones. The parietal bones were flapped out to the sides with a rongeur, and the brain became accessible. After removing the cerebellum with a spatula, the forebrain was scooped out and placed into ~10 mL cold isolation buffer (IB; for details, see Table S2 in Supplement).

### 2.2. Isolation of Mitochondria

The brain mitochondria were isolated following—with some modifications—the procedure of Kristian [22] using differential centrifugation. The isolation (IB, Table S2) and experimental buffers (EB, Table S3) were derived from Holmuhamedov et al. [23]. Throughout the isolation process, the IB used was continuously kept on ice.

After placing the brain in the IB, the tissue was rinsed clear of blood by adding more IB and pipetting the IB up and down with a plastic pipette. After weighing the brain, it was placed in a beaker with ~3 mL IB. Using scissors, the tissue was minced into ~1-mm pieces and transferred to a homogenizing vessel. Ten ml of IB was added, and the pestle was passed up and down 8 to 10 times in the homogenizer until the homogenate was smooth and uniform. The homogenate was transferred into a 50-mL centrifuge tube and centrifuged (1300× *g* at 4 °C) for 3 min. The supernatant was then carefully transferred into a new tube placed on ice. The pellet was gently dislodged and homogenized in 5 mL of IB using a plastic transfer pipette. The new homogenate was centrifuged again at 1300× *g* and 4 °C for 3 min. The resulting supernatant was combined with the supernatant collected from the first centrifugation. The pooled supernatant was centrifuged at 21,000× *g* and 4 °C for 10 min. In the meantime, the Percoll^®^ (Cytiva, Marlborough, MA, USA) gradient was prepared as follows [22]:

Percoll^®^ (24%, 7.4 mL) was added into a centrifugation tube. To ensure careful insertion, 3 mL of 40% Percoll^®^ was first added into a separate tube with a volumetric pipette. The 3 mL of 40% Percoll^®^ was collected with a transfer pipette and inserted into the 24% Percoll^®^ such that the tip touched the bottom of the tube at a slight angle. The 40% Percoll^®^ was then introduced slowly to the bottom to create two distinct layers in the solution.

After centrifugation, the pellet was dislodged, resuspended in 3.5 mL of 15% Percoll^®^ using a plastic transfer pipette, and slowly layered above the 24% Percoll^®^; the introduction of the top layer was begun by leaning the tip of the pipette against the tube wall close to the surface of the 24% Percoll^®^ and adding slowly so that distinct layers would appear. To prepare one rat forebrain, two gradients in one tube each were prepared each time, so that one of the two could be used as centrifugation control.

Using the second tube as a balance tube, the Percoll^®^ gradients (one of them containing the homogenate) were centrifuged at 30,700× *g* and 4 °C for 8 min using slow acceleration (45 s from 0 to 500 rpm followed by normal acceleration) and slow deceleration (no brakes), to redistribute the tissue material into three major bands. When finished, the material at the top of the gradient—containing mostly myelin—as well as the underlying layer—containing mostly synaptosomes—was removed with an adjustable 1-mL volumetric pipette. Using the 1-mL volumetric pipette, the Percoll^®^ solution containing material accumulating at the interface between the 24% and 40% Percoll^®^ solution—which is enriched by non-synaptic mitochondria—was collected and added into a separate centrifuge tube. After adding ~6 mL of IB into this tube, it was centrifuged at 16,700× *g* and 4 °C for 10 min. The mitochondria would then collect at the bottom of the tube as a loose pellet. The supernatant was poured off carefully, the resulting pellet was resuspended in ~1 mL of IB, and another ~10 mL of buffer was added. The mitochondrial fraction was centrifuged again at 6900× *g* and 4 °C for 10 min. The supernatant was decanted, and any remaining solution was removed from the wall of the centrifuge tube. The pellet was gently resuspended in 0.25 mL of IB, put into a 1.5-mL tube, and kept on ice.

### 2.3. Determination of Mitochondrial Protein Concentration

After the isolation process, the mitochondrial protein concentration was determined using the Bio-Rad Protein Assay—Standard Procedure for Microtiter Plates based on the dye-binding method of [24]: absorbance was measured in a spectrophotometer (BioTek Instruments, Inc., Winooski, VT, USA) at 595 nm. Mitochondria sample concentration was determined by plotting the standard absorbances (after subtracting the blank absorbance) to the standard concentrations and calculating a regression line by which the mitochondrial sample absorbance could be transferred into a concentration (Figure S1).

### 2.4. Injuring Mitochondria

#### 2.4.1. In Vitro Injury with H_2_O_2_

One approach to injure mitochondria was to expose isolated mitochondria to H_2_O_2_ for a limited time. Preliminary data from our laboratory had suggested that a concentration of 200 µM for 10 min would be sufficient to decrease mitochondrial viability by 50%. To do so, the mitochondrial sample was moved from the ice-cold surrounding to room temperature (RT), and H_2_O_2_ was added to result in 200 µM H_2_O_2_ in mitochondrial solution. The mitochondrial sample was then gently shaken for 10 min on a microplate shaker at 500 rpm. A control mitochondrial sample was incubated the same way, but without H_2_O_2_ added to the mitochondria.

#### 2.4.2. In Vivo Injury by Asphyxial CA

The other approach to injure mitochondria was to induce an in vivo asphyxial CA. For this purpose—after induction of general anesthesia (see above)—CA was achieved following a protocol modified from Katz et al. [25]. After preparation and a stabilization phase of 30 min, 3 mg×kg^−1^ of the neuromuscular blocker rocuronium bromide (Hospira, Inc., Lake Forest, IL, USA) was given intravenously. Then, 2 min later, the ventilation was stopped, thus inducing asphyxia. This reliably resulted in a CA after ~3 min, which was defined as a systolic blood pressure less than 20 mmHg. Asphyxial CA was maintained for 15 min. Sham control rats underwent the same procedure, but without sustaining asphyxia and CA. Immediately after the defined time of asphyxia, the anesthetized animal was euthanized by decapitation and the mitochondria isolated.

### 2.5. Treatment with P188

#### 2.5.1. After H_2_O_2_-Induced Injury In Vitro

The treatment with P188 after H_2_O_2_-induced injury occurred in two different ways: P188 was applied in a final concentration of 250 µM by diluting the mitochondria sample 1:2 with 500 µM P188 in EB, hereby slowing the reaction of H_2_O_2_. After diluting, mitochondria were either placed back on ice or gently shaken at 500 rpm at RT for 10 more min until placed on ice as well. Control mitochondria underwent a 1:2 dilution with plain EB and were also placed on ice immediately or after 10 min, respectively.

#### 2.5.2. After Asphyxial CA-Induced Injury In Vivo

The mitochondrial sample was diluted to 2 mg mL^−1^ protein concentration with ice-cold EB. Then, 250 µM P188 was applied by diluting 1:2 with 500 µM P188 in EB. The sample was shaken at RT for 10 min and placed back on ice. Control mitochondrial samples diluted with plain EB were either prepared the same way or put on ice immediately.

### 2.6. Assessment of Mitochondrial Function Parameters

We used three methods to assess mitochondrial viability: ATP synthesis, O_2_ consumption, and CRC. They have been described before [9,26,27,28].

#### 2.6.1. ATP Synthesis Assay

**Figure A1:**
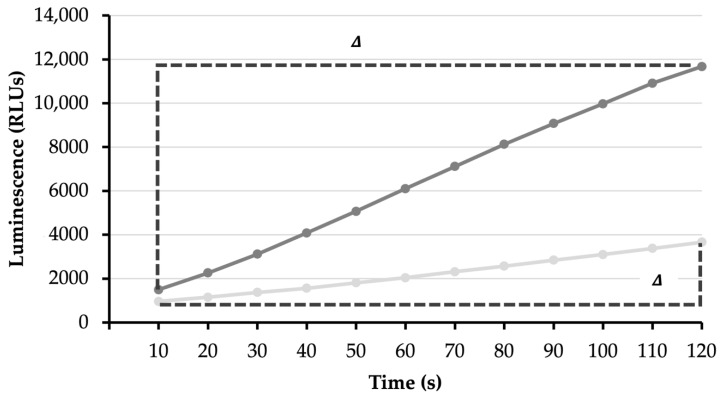
Mitochondrial adenosine triphosphate (ATP) synthesis is measured by the reaction of ATP with luciferase. With higher ATP synthesis, luciferase produces more light, which is measured by an increase in relative luminescence units (RLUs; compare dark grey to light grey lines). The difference (Δ) in RLUs over time determines the rate of ATP synthesis.

The ATP Assay Buffer (for details see Table S4)—containing adenosine diphosphate (ADP), diadenosine pentaphosphate, luciferin (Tocris Bioscience, Minneapolis, MN, USA), and luciferase (Firefly-Enzyme; G-Biosciences, St. Louis, MO, USA)—allows functional mitochondria to produce ATP, resulting in bioluminescence measured by a luminometer (GloMax^®^ 20/20, Promega Corporation, Madison, WI, USA). Thus, the more ATP is produced, the more luminescence will be measured, indicating higher mitochondria viability (Figure A1).

A clear Eppendorf tube was filled with 500 µL of ATP assay buffer and placed in the luminometer. First, 10 µL of mitochondrial suspension, then 5 µL of combined 0.5 M sodium glutamate/0.5 M disodium malate (complex I substrates) or 5 µL of 0.5 M succinate (complex II substrate) with 5 µL of 0.5 mM rotenone—depending on which complexes were tested—were added, starting the reaction. The rate of ATP produced was then calculated in µmol min^−1^ mg^−1^ by the rate of increase in luminescence in relation to a standard curve derived from known ATP concentrations ranging from 2.5 to 200 µM.

#### 2.6.2. O_2_ Consumption Assay

**Figure A2:**
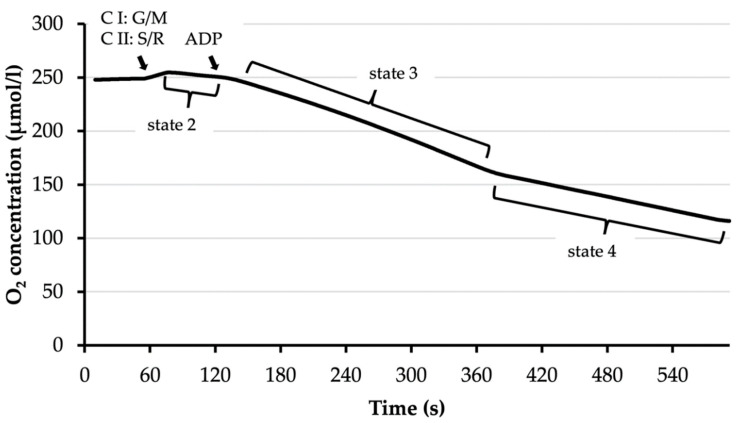
Mitochondrial oxygen (O_2_) consumption can be divided into different states. State 2 occurs when substrates of the respiratory chain are present except for adenosine diphosphate (ADP). The substrates to fuel into complex I (C I) are glutamate/malate (G/M), while complex II (C II) is propelled by succinate (S) and isolated through blocking complex I with rotenone (R). State 3 is then achieved by adding ADP, which enables the adenosine triphosphate (ATP) synthase. When ADP is completely consumed, O_2_ consumption switches into state 4. Respiratory control index (RCI) = state 3/state 4.

To use this method, a Respirometer Chamber (Strathkelvin Instruments Limited, North Lanarkshire, Scotland) with a Clark-type electrode was used (Figure S2). EB and 70 µL mitochondria were introduced into the 350 µL chamber, and the plunger was inserted to close it. The O_2_ concentration in the mitochondrial suspension was measured for 60 s. Then, either 3.5 µL of combined 1 M glutamate/1 M malate solution (complex I substrates) or 3.5 µL of 1 M succinate (complex II substrate) with 3.5 µL of 1 mM rotenone—depending on which complexes were tested—were added. This activated state 2 of mitochondrial respiration. After another 60 s, 3.5 µL of 25 mM ADP was added. This started the state 3 respiration, which is characterized by higher O_2_ consumption, i.e., a faster drop in O_2_ concentration in the respirometer chamber. The O_2_ concentration was observed until the O_2_ consumption decreased, signaling the beginning of state 4. From this point, the O_2_ concentration was monitored for another minute before the recording was stopped. Regression lines in the different states were calculated to identify the rate of O_2_ consumption in state 3 and state 4 (Figure A2) and to calculate the respiratory control index (RCI) as O_2_ consumption in state 3 divided by O_2_ consumption in state 4.

#### 2.6.3. CRC Assay

**Figure A3:**
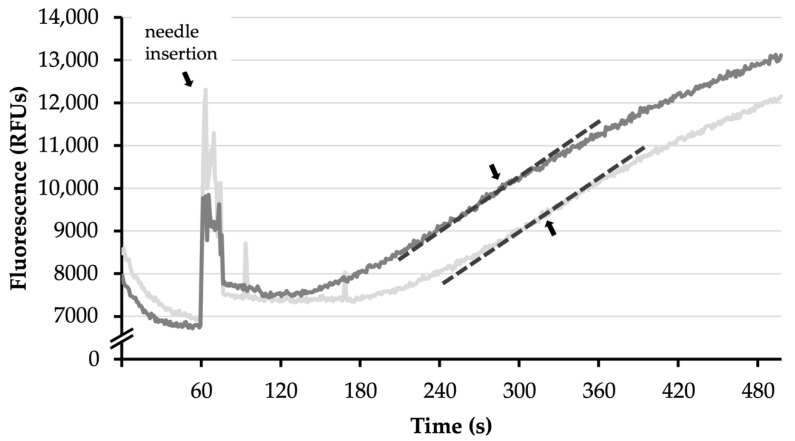
Calcium retention capacity (CRC) is defined as the maximal amount of calcium (Ca^2+^) that can be kept inside mitochondria. With the insertion of the needle into the cuvette, calcium chloride (CaCl_2_) is pumped at a continuous rate into the mitochondrial suspension. Extramitochondrial Ca^2+^ binds Calcium Green^TM^. This results in increased fluorescence measured in relative fluorescence units (RFUs). The highest slope (dotted lines) indicated the moment at which mitochondrial permeability transition (MPT) occurred in most mitochondria. This allowed calculation of the amount of Ca^2+^ infused into the mitochondrial suspension until this point.

Similar to the endoplasmic reticulum, mitochondria are able to retain calcium (Ca^2+^) ions in order to maintain a controlled cytoplasmatic Ca^2+^ concentration [29]. Mitochondrial Ca^2+^ retention, though, can lead to mitochondrial swelling proportional to the Ca^2+^ retained. If too much Ca^2+^ is retained, the mPTP will open and release the Ca^2+^. The CRC assay works by using Calcium Green^TM^ (Thermo Fisher Scientific, Waltham, MA, USA), a Ca^2+^-binding fluorescent dye which cannot pass through the mitochondrial membrane. External Ca^2+^ is added gradually to the solution and is taken up and retained by the mitochondria. When the mPTP opens, the released Ca^2+^ will bind to the dye, resulting in a fluorescent reaction which can be timed and quantified with a fluorescence spectrophotometer (Horiba, Piscataway, NJ, USA) at 506 nm excitation and 532 nm emission. First, 450 µL of 100 nM Calcium Green EB and 50 µL mitochondria were added into a 1-mL cuvette. Then, either 5 µL of combined 1 M glutamate/1 M malate solution (complex I substrates) or 5 µL of 1 M succinate (complex II substrate) and 5 µL of 1 mM rotenone—depending on which complexes were tested—were added. After 60 s, the needle attached to the syringe filled with 1 mM calcium chloride (CaCl_2_) in no-phosphate EB was inserted through a small hole in the lid, and the infusion started at 10 µL min^−1^ (Figure S3). After a short spike, the recording typically showed a steady state, in which all the Ca^2+^ infused was immediately taken up and retained by the mitochondria (Figure A3). When MPT occurred, the Ca^2+^ released bound to the Calcium Green^TM^ dye, and the resulting fluorescence increased. To determine CRC, the time point of the fastest increase, i.e., the steepest slope, was measured, when MPT was occurring in most mitochondria (Figure A3). This allowed the calculation of CRC as the amount of Ca^2+^ in µmol mg^−1^ infused into the mitochondrial suspension until this point.

### 2.7. Data Analysis and Statistics

Independently of the assay used to assess mitochondrial function, all resulting data were tested for normality with the Shapiro–Wilk test and for homoscedasticity (homogeneity of variance) with the Levene’s test. If both tests were passed (*p* > 0.05), data analyses were conducted through parametric testing. If one or both of them failed (*p* < 0.05), non-parametric testing was performed.

For parametric testing, one-way ANOVA was performed to compare the means of the different groups. If a statistically significant difference (*p* < 0.05) was found, the Student–Newman–Keuls method was applied as a post-hoc test to detect between-group differences.

If only the Levene’s test but not the Shapiro–Wilk test failed, data were analyzed with tests which do not require homoscedasticity. These were namely the Welch’s one-way ANOVA and the Games–Howell post-hoc test, the latter one only being used if significant differences (*p* < 0.05) were detected.

For data that failed the Shapiro–Wilk test, the non-parametric Kruskal–Wallis test was performed. If significant differences (*p* < 0.05) were found, the Wilcoxon rank-sum test was applied for post-hoc testing.

Graphically, statistically significant differences (*p* < 0.05) are indicated by brackets or *. Since not all data fulfilled the criteria for parametric testing, results are presented as box plots to portray the data distribution accurately.

All data were analyzed using R [30] with the packages “car” [31], “dplyr” [32], “ggoubr” [33], “agricolae” [34], and “userfriendlyscience” [35].

## 3. Results

### 3.1. Isolated Mitochondria after H_2_O_2_-Induced Injury

Isolated mitochondria were incubated with 200 µM H_2_O_2_ for 10 min before being diluted with or without 250 µM P188. Thereafter, mitochondrial function parameters for complex I and II substrates were assessed, specifically ATP synthesis, O_2_ consumption, and CRC. The results were stratified for the four groups that resulted from the injuring and treatment process (control, control + P188, injured, injured + P188).

#### 3.1.1. ATP Synthesis after H_2_O_2_-Induced Injury

**Figure 1:**
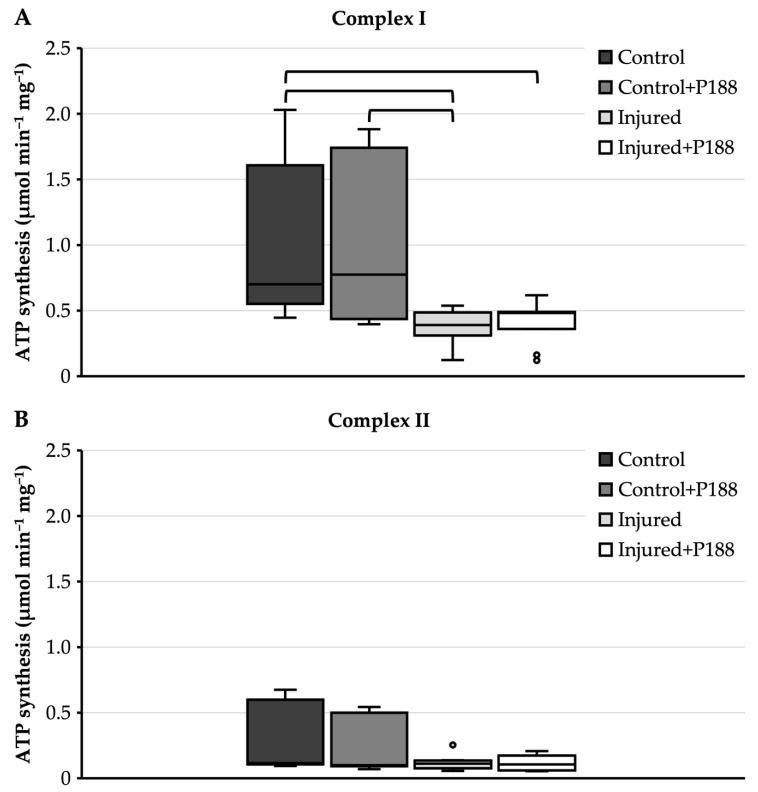
Adenosine triphosphate (ATP) synthesis (µmol⋅min^−1^ mg^−1^) in hydrogen peroxide (H_2_O_2_)-injured, isolated mitochondria for complex I (**A**) and II (**B**) substrates in control, control + Poloxamer (P)188, injured, and injured + P188 mitochondria. Boxplots show 25th, 50th, and 75th percentile; whiskers represent minimum and maximum. Statistical significance (*p* < 0.05) denoted by brackets; complex I: *n* = 5, complex II: *n* = 5.

For complex I substrates, ATP synthesis of control mitochondria showed a median of 0.7 µmol⋅min^−1^⋅mg^−1^ with an interquartile range from 0.55 to 1.61 µmol min^−1^ mg^−1^ (Figure 1A). When adding P188 to the control, ATP synthesis remained fairly equal to the control without P188, with no significant differences (Figure 1A). The ATP synthesis of injured mitochondria without P188 was significantly impaired compared to the control mitochondria, with a median of 0.39 µmol min^−1^ mg^−1^ and an interquartile range from 0.31 to 0.49 µmol⋅min^−1^ mg^−1^ (Figure 1A). This was, again, independent of P188 treatment as P188 did neither increase nor decrease ATP synthesis in injured mitochondria. However, although the ATP synthesis of mitochondria treated with P188 appears to differ in control mitochondria compared to injured mitochondria, with the medians being 0.77 and 0.48 µmol⋅min^−1^ mg^−1^, respectively, the difference is not statistically significant (Figure 1A).

For complex II substrate, ATP synthesis of control mitochondria showed a median of 0.12 µmol min^−1^ mg^−1^ with an interquartile range from 0.11 to 0.6 µmol min^−1^ mg^−1^ (Figure 1B). The addition of P188 to control mitochondria did not lower ATP synthesis significantly (Figure 1B). Median ATP synthesis of injured mitochondria was not markedly different compared to control mitochondria; however, the interquartile range was strongly reduced, ranging from 0.08 to 0.14 µmol min^−1^ mg^−1^ (Figure 1B). The same applies for injured mitochondria with added P188, where the interquartile range of ATP synthesis ranged from 0.06 to 0.17 µmol min^−1^ mg^−1^ (Figure 1B). However, the difference in ATP synthesis of injured mitochondria compared to control mitochondria was not significant, and this was independent of the addition of P188 (Figure 1B).

#### 3.1.2. O_2_ Consumption after H_2_O_2_-Induced Injury

**Figure 2:**
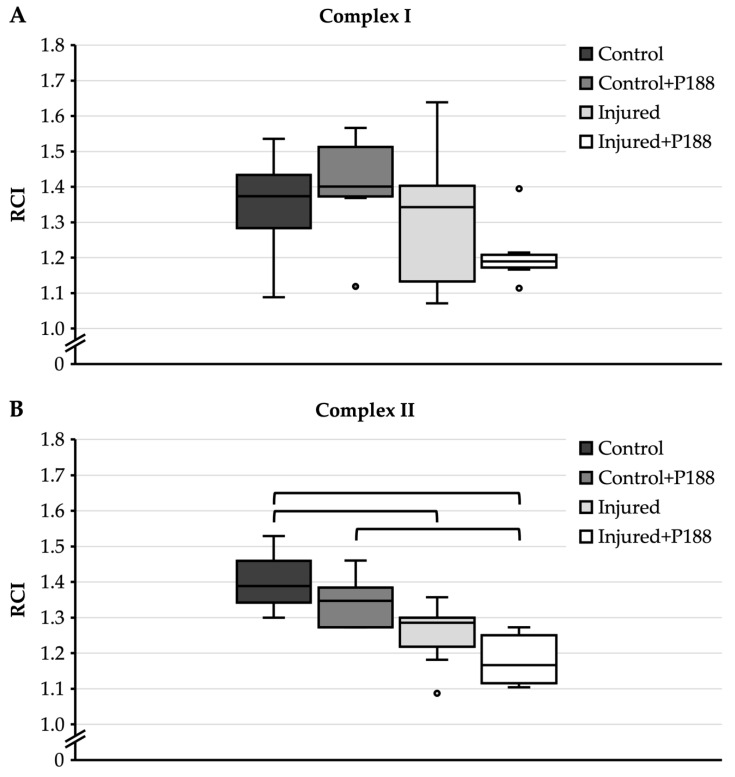
Respiratory control index (RCI) of oxygen (O_2_) consumption in hydrogen peroxide (H_2_O_2_)-injured, isolated mitochondria for complex I (**A**) and II (**B**) substrates in control, control + Poloxamer (P)188, injured, and injured + P188 mitochondria. Boxplots show 25th, 50th, and 75th percentile; whiskers represent minimum and maximum. Statistical significance (*p* < 0.05) denoted by brackets; complex I: *n* = 6, complex II: *n* = 5.

For complex I substrates (Figure 2A), control mitochondria had a median RCI of 1.37 with an interquartile range from 1.28 to 1.43. The addition of P188 to control mitochondria resulted in a median RCI of 1.4 with an interquartile range from 1.37 to 1.51. The median RCI of injured mitochondria without P188 treatment was 1.34, the interquartile range from 1.13 to 1.4 extended markedly lower than the interquartile range of control mitochondria. Injured mitochondria treated with P188 had a median RCI of 1.19 and an interquartile range from 1.17 to 1.21. However, there was no statistically significant difference among the groups.

For complex II substrate (Figure 2B), control mitochondria showed a median RCI of 1.39 with an interquartile range from 1.34 to 1.46. The addition of P188 to control mitochondria did not significantly decrease RCI. The RCI of injured mitochondria without P188 treatment was reduced to 1.29, and this was significantly different compared to control mitochondria without P188 treatment. The addition of P188 to injured mitochondria again led to a decrease in RCI to 1.18, and this was significantly different compared to control mitochondria with and without P188 treatment.

#### 3.1.3. CRC after H_2_O_2_-Induced Injury

**Figure 3:**
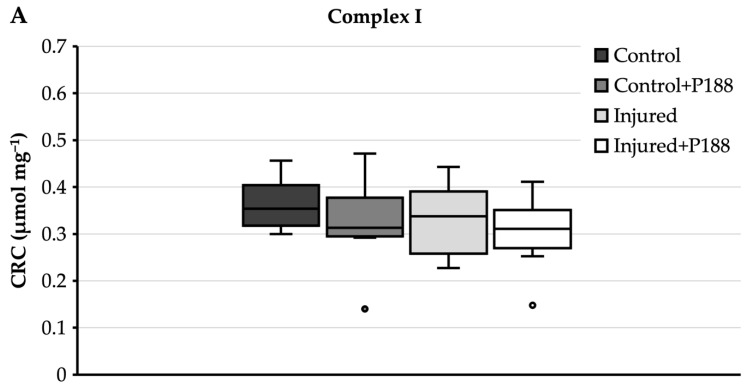

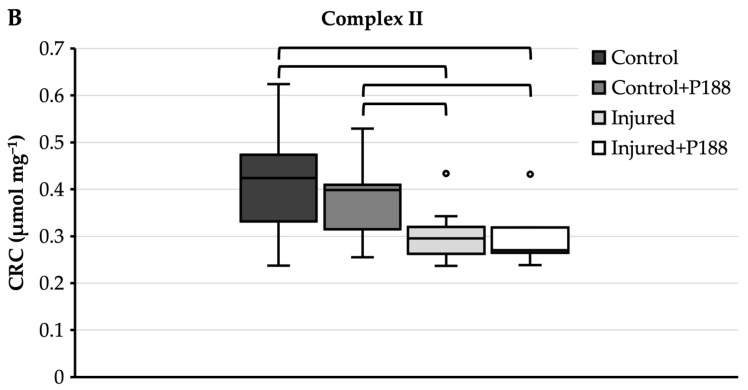
Calcium retention capacity (CRC) (µmol⋅mg^−1^) in hydrogen peroxide (H_2_O_2_)-injured, isolated mitochondria for complex I (**A**) and II (**B**) substrates in control, control + Poloxamer (P)188, injured, and injured + P188 mitochondria. Boxplots show 25th, 50th, and 75th percentile; whiskers represent minimum and maximum. Statistical significance (*p* < 0.05) denoted by brackets; complex I: *n* = 4, complex II: *n* = 5.

For complex I substrates (Figure 3A), control mitochondria had a median CRC of 0.35 µmol mg^−1^ and an interquartile range from 0.32 to 0.4 µmol mg^−1^. Neither the addition of P188 to control mitochondria, nor injury with H_2_O_2_, nor P188 treatment of H_2_O_2_ injury changed this significantly.

For complex II substrate (Figure 3B), control mitochondria showed a median CRC of 0.42 µmol mg^−1^ and an interquartile range from 0.33 to 0.47 µmol mg^−1^ (Figure 3B). With a median of 0.4 µmol mg^−1^, the CRC of P188-treated control mitochondria was not significantly lower than control mitochondria without P188. Injured mitochondria, however, showed a median CRC of 0.3 µmol mg^−1^ and 0.27 µmol mg^−1^ without and with P188, respectively, and these were significantly lower than in control mitochondria, independently of treatment with P188.

### 3.2. Comparison of Different Exposure Times to RT

Throughout the course of the experiments, two different injuring regimens were executed. The main setting has been presented above and consisted of 10 min of exposure to H_2_O_2_ at RT and further 10 min RT after dilution with or without P188, leading to a total 20 min of RT exposure (20 min RT). In the second setting, the isolated mitochondria were immediately placed on ice after the 10 min of H_2_O_2_-induced injury at RT (10 min RT). The two settings were compared to examine the effects of prolonged exposure to RT on the isolated mitochondria. The results within the 20 min RT condition have been presented above.

**Figure A4:**
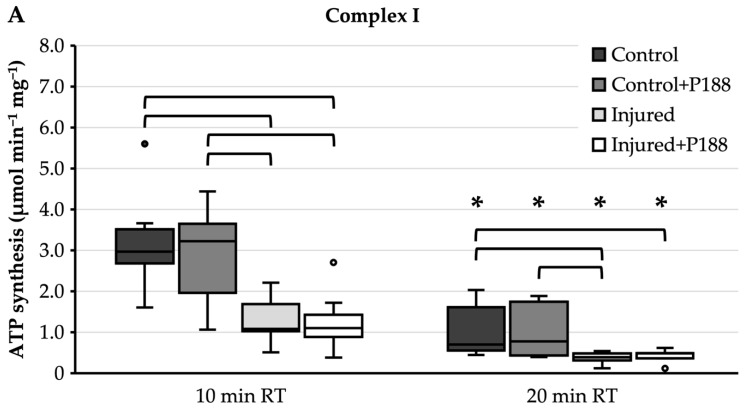

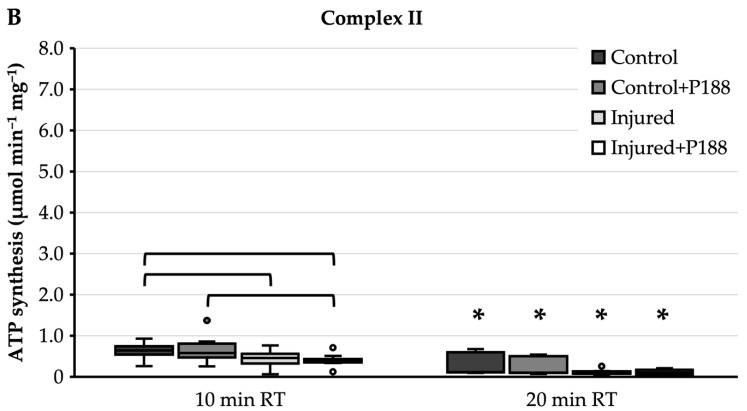
Comparison of adenosine triphosphate (ATP) synthesis (µmol⋅min^−1^⋅mg^−1^) between differently treated hydrogen peroxide (H_2_O_2_)-injured, isolated mitochondria for complex I (**A**) and II (**B**) substrates in control, control + Poloxamer (P)188, injured, and injured + P188 mitochondria. Condition is defined by total time of exposure of isolated mitochondria to room temperature (RT). Boxplots show 25th, 50th, and 75th percentile; whiskers represent minimum and maximum. Statistical significance (*p* < 0.05) within one condition was denoted by brackets; statistical significance (*p* < 0.05) between the same groups of different conditions was denoted by *; complex I: 10 min RT: *n* = 7, 20 min RT: *n* = 5; complex II: 10 min RT: *n* = 7, 20 min RT *n* = 5.

After 10 min RT, median ATP synthesis of control mitochondria for complex I substrates was at 3 and 3.2 µmol min^−1^ mg^−1^ without and with P188, respectively (Figure A4A in the Appendix A). Injured mitochondria showed significantly lower ATP synthesis than control mitochondria at a median of 1.1 µmol min^−1^ mg^−1^ without as well as with P188.

For complex II substrate, median ATP synthesis after 10 min RT was at 0.6 µmol min^−1^ mg^−1^ in control mitochondria, independently of the treatment with P188 (Figure A4B). Median ATP synthesis of injured mitochondria without P188 was at 0.5 µmol min^−1^ mg^−1^ and with that slightly but significantly lower than control mitochondria without P188. At a median of 0.4 µmol min^−1^ mg^−1^, ATP synthesis of P188-treated injured mitochondria was significantly lower than control mitochondria, independently of the treatment with P188.

ATP synthesis for both complex I and complex II substrates was significantly decreased after 20 min RT compared to 10 min RT incubation in all four treatment groups, respectively (Figure A4).

### 3.3. Isolated Mitochondria after Asphyxial CA-Induced Injury

In addition to the in vitro application of H_2_O_2_, an in vivo injuring strategy was applied as well to account for the missing intracellular but extra-mitochondrial processes during ischemia. For this purpose, cerebral ischemia was induced in rats for 15 min by asphyxial CA; sham rats were ventilated for the same duration without undergoing asphyxia. The anesthetized animals were then euthanized and the forebrain mitochondria isolated. Subsequently, the mitochondria were either diluted with 250 µM P188, 500 µM P188, or only with EB (control), and kept at RT for 10 min, or kept on ice completely, before assessing O_2_ consumption.

**Figure 4:**
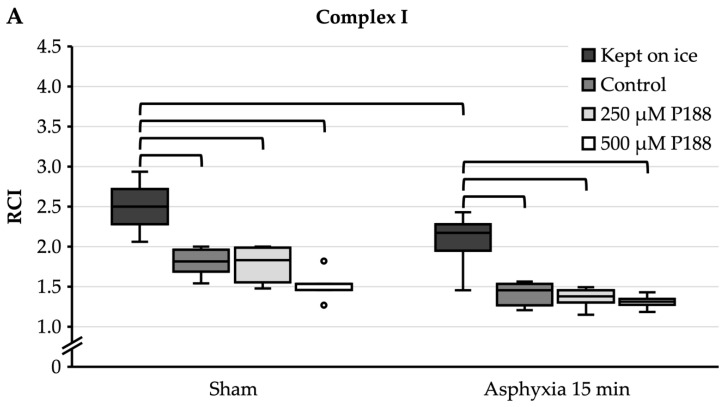

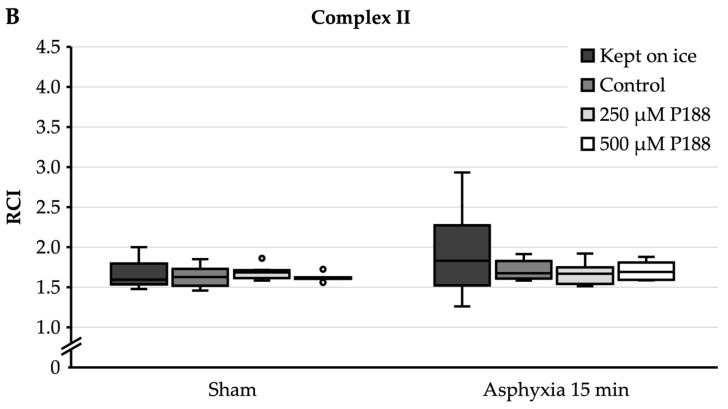
Comparison of respiratory control index (RCI) of oxygen (O_2_) consumption in cardiac arrest (CA)-injured, isolated mitochondria for complex I (**A**) and II (**B**) substrates in mitochondria continuously kept on ice, control, treated with 250 µM Poloxamer (P)188, and treated with 500 µM P188. Prior to mitochondria isolation, asphyxial CA had been induced in the rats with an asphyxial time of 15 min. Sham rats were ventilated throughout anesthesia without induction of asphyxia. Boxplots show 25th, 50th, and 75th percentile; whiskers represent minimum and maximum. Statistical significance (*p* < 0.05) denoted by brackets; complex I: *n* = 3; complex II: *n* = 3.

For complex I substrates (Figure 4A), sham mitochondria that were completely kept on ice showed a median RCI of 2.5. In mitochondria kept on ice, asphyxia of 15 min significantly decreased RCI to 2.2. Under sham conditions, control, 250 µM P188, and 500 µM P188 mitochondria showed a significantly lower RCI, between 1.5 and 1.8, compared to sham mitochondria that were kept on ice. Similarly, in the 15 min asphyxia condition, the RCI of control, 250 µM P188, and 500 µM P188 mitochondria was significantly reduced to between 1.3 and 1.5 compared to mitochondria kept on ice. Furthermore, with 15 min of asphyxia, a seeming decrease in RCI in control, 250 µM P188, and 500 µM P188 mitochondria compared to the sham condition was not significant.

For complex II substrate (Figure 4B), sham mitochondria that were completely kept on ice showed an RCI of 1.6. Asphyxia of 15 min led to an RCI of 1.8, with no significant differences being found. Control, 250 µM P188, and 500 µM P188 mitochondria showed no significant differences to each other or to mitochondria kept on ice, neither within their condition nor outside.

## 4. Discussion

The triblock copolymer P188 has been shown to rescue a variety of tissues and cell types from I/R injury in different settings [9,10,11,12,13,14,15,16,17,18,19,36,37,38]. More recently, profound improvements in mitochondrial function through P188 after I/R injury have become apparent [9,10,11,12,18,39,40], suggesting possible direct interactions of P188 with mitochondria, specifically by stabilizing their membrane.

In the present study, oxidative stress was induced in vitro as well as in vivo in rat isolated forebrain mitochondria. H_2_O_2_ significantly impaired ATP synthesis for complex I substrates as well as O_2_ consumption and CRC for complex II substrate. Asphyxia of 15 min significantly impaired O_2_ consumption for complex I substrates. Additionally, the results of both the in vivo and in vitro experiments show that RT has a harmful effect on isolated mitochondria and may be concealing differences between different mitochondrial treatment groups. Finally, independently of the injury mechanism, P188 did not show any significant effects on mitochondria and was not able to alleviate mitochondrial impairment after simulation of oxidative stress.

### 4.1. Methods of Injuring Isolated Mitochondria

#### 4.1.1. In Vivo Injury

P188′s superb protective effects after I/R injury have been recreated in various experimental settings [9,10,11,12,13,14,15,16,17,18,19,36,37,38]. Therefore, injuring mitochondria in a way that mimics the patho-physiological processes of I/R in living cells was the premise to show any direct effects of P188 on mitochondria. The induction of global and, specifically, cerebral ischemia by asphyxial CA of 15 min in rats in vivo represents a realistic injury. Brain mitochondria were isolated immediately after ischemia, but without yet introducing reperfusion. Only when isolated, mitochondria would then be exposed to RT and P188 at the same time to simulate reoxygenation as P188 seems to work optimally when applied at the beginning of reperfusion [9]. By isolating mitochondria first before adding P188, it was ensured that no cellular processes could detract from P188′s potential direct interaction with mitochondria.

While isolating mitochondria before introducing IR is an alternative approach, the basic model of I/R in vivo has already been described in the literature. In fact, Bartos et al. pig isolated heart mitochondria after 45 min ischemia and 4 h reperfusion and observed a drop in O_2_ consumption from an RCI of 8 to 4 for complex I substrates and from 3.5 to 2 for complex II substrate [9]. This reduction of approximately 50% is slightly higher than the reduction in RCI in the present study; however, the ischemic duration was three to four times as long [9]. Matsuura et al., too, used a pig model in which they examined the effects of global ischemia after ventricular fibrillation for 19 min or for 15 min with 4 min of reperfusion (cardiopulmonary resuscitation) [26]. Here, cardiac as well as cerebral mitochondria were examined: compared to no ischemia, O_2_ consumption of cardiac mitochondria was only significantly reduced for complex II substrate after ischemia both with and without reperfusion [26]. Interestingly, O_2_ consumption of cerebral mitochondria was significantly reduced for both complex I and II substrates after ischemia and independently of reperfusion, showing the high susceptibility of brain mitochondria to ischemic damage [26]. In this case, the time of ischemia and the reduction in O_2_ consumption approximately match the ones used and achieved in the present study [26]. However, this has to be viewed with the caveat that these results were achieved in pigs and not in rats [9,26].

#### 4.1.2. In Vitro Isolated Mitochondria Injury

Injuring mitochondria after isolation proves to be favored when trying to study their specific behavior in I/R injury, as it allows for more targeted alterations in mitochondrial processes. This becomes evident when looking at the vast literature on this topic [41,42,43,44,45,46,47,48,49,50,51,52]. For instance, anoxia/reoxygenation is an established method to simulate I/R injury in isolated mitochondria, similar to in vivo and ex vivo settings as well as in vitro cell cultures [44,45,49,50]. Ozcan et al. sealed rat isolated cardiac mitochondria in an airtight multichannel chamber, letting the mitochondria consume the O_2_ within 5 min to create anoxic conditions [49]. As soon as the O_2_ was completely consumed, 20 min of anoxia was timed before exposing the mitochondria to RT for another 20 min [49,50]. This procedure led to significantly decreased ATP synthesis and O_2_ consumption [49]. Interestingly, the treatment of mitochondria with ROS scavengers significantly attenuated the anoxic damage, hinting towards the detrimental effects of ROS on mitochondria that were shown in the present study as well [49]. In contrast, Korge et al. used a closed cuvette to conduct their rabbit isolated heart mitochondria experiments in. Instead of waiting for the mitochondria to consume the O_2_, they actively directed nitrogen into the buffer with the mitochondria through a hole in the cuvette cover [45]. To reoxygenate, they then simply exchanged the nitrogen for O_2_ [45]. Another study describes generating anoxia in mouse isolated heart mitochondria by introducing Argon into the buffer until the O_2_ concentration decreased below <15 nmol O_2_ mL^−1^ [44]. Anoxia was then maintained for 6 min before adding air-saturated incubation buffer to the mitochondria for another 3 min to simulate reperfusion, again, leading to decreased ATP synthesis, O_2_ consumption, and also CRC compared to a time control [44].

As described above, Ca^2+^ overload occurs during I/R and contributes to the opening of mPTPs [5,7,53]. By adding Ca^2+^ to isolated mitochondria, these circumstances can be replicated fairly easily [43,46,51]. For instance, Crestanello et al. added Ca^2+^ in concentrations of 0.1 or 0.5 µM to rat isolated heart mitochondria and were able to observe that increasing Ca^2+^ concentrations uncoupled mitochondrial oxidative phosphorylation [43]. In a study focusing specifically on the role of complex II in the production of ROS, 50 µM of Ca^2+^ induced MPT as well as increased the production of H_2_O_2_, and this was inhibited by pre-incubating the rabbit isolated cardiac mitochondria with ethylene glycol-bis(β-aminoethyl ether)-*N*,*N*,*N*′,*N*′-tetraacetic acid to chelate Ca^2+^ [46]. Another study examining a radical scavenger to inhibit MPT found that the addition of Ca^2+^ (30 to 300 µM) to rat isolated brain mitochondria caused increased ROS production in a dose-dependent manner [51]. This shows that the same mechanism used in the present study to calculate the CRC, namely the activation of MPT through the addition of exogenous Ca^2+^, can be used to simulate one particular aspect of I/R in mitochondria. Furthermore, it is interesting to observe that Ca^2+^ overload also contributes to ROS production as this could mean that Ca^2+^ overload happens more upstream in the reaction chain of I/R.

Lastly, ROS as a well-investigated component of I/R injury have been used to simulate the latter in isolated mitochondria [41,47,48,50,51,52]. This approach was used already in 1986 when Malis and Bonventre added hypoxanthine, xanthine oxidase, and ferrous chloride to rat isolated renal mitochondria, resulting in a decrease in mitochondrial respiration for both complex I and II substrates [48]. Interestingly, they also added Ca^2+^ at a concentration of 30 nmol mg^−1^ mitochondrial protein and, while this showed no impact on mitochondrial function by itself when combined with ROS, it significantly decreased mitochondrial respiration even further when complex I substrates were given compared to only ROS [48]. Similarly, Makazan et al. used xanthine and xanthine oxidase for 1, 2, or 3 min on rat isolated heart mitochondria, resulting in reduced mitochondrial respiration in a time-dependent manner [47]. Although the amount of ROS produced was not quantified, these findings once more exemplify the harmful effects of ROS on mitochondria, which were again confirmed in the present study.

Instead of combining an enzyme and its substrate to create ROS, Makazan et al. also added H_2_O_2_ as a ROS itself to the mitochondria [47]. This was done for 3 min at concentrations of 10, 20, and 30 µM, again causing decreased mitochondrial respiration, this time in a concentration-dependent manner [47]. H_2_O_2_ was also used by the aforementioned Ozcan et al. study at a concentration of 100 µM for 30 min, causing the rat isolated heart mitochondria to swell by approximately 33% [50]. When using concentrations between 10 and 300 µM, they were able to show that H_2_O_2_ inhibits ATP production in a concentration-dependent manner, with the strongest decrease being from 100 to 200 µM [50]. On their rabbit isolated heart mitochondria, Chen and Lesnefsky, too, used 100 µM of H_2_O_2_ but for only 5 min [41]. Nevertheless, they were able to observe significant mitochondrial swelling [41]. This could mean that H_2_O_2_-induced damage to isolated mitochondria is not time-dependent. However, H_2_O_2_ has been used at even higher concentrations, namely 2 mM H_2_O_2_ for 5 min in rat isolated cardiac mitochondria or 3 mM H_2_O_2_ for 7 min on rat isolated brain mitochondria [51,52]. For the present study, 200 µM H_2_O_2_ for 10 min as a setting for I/R simulation was chosen as it offered a significant decrease in mitochondrial function parameters with the potential for damage to be further exacerbated or, rather, ameliorated.

### 4.2. Effect of P188 on Mitochondria

As mentioned above, Bartos et al. were able to show in a porcine model of myocardial infarction that, when applied directly upon reperfusion, P188 is able to not only decrease serum troponin I and infarct size but also to improve cardiac mitochondrial function assessed by mitochondrial respiration and CRC [9]. These results led to the hypothesis that mitochondria might be directly affected by P188 and consequently led to the present study. Bartos et al., however, were not the only ones that specifically examined mitochondrial function after treating animals or cell cultures with P188 [10,11,12,18,39,40,54,55].

To simulate I/R in cell cultures, Shelat et al. used oxygen-glucose deprivation (OGD) for 30, 45, or 60 min with subsequent 48 h of reoxygenation on rat hippocampal neurons [40]. They then treated the cells with P188 in concentrations of 0.3, 3, 30, or 100 µM and for 1, 6, 12, 15, 18, or 24 h after OGD [40]. P188 was able to rescue hippocampal neurons from apoptosis after OGD, and this was done optimally at a concentration of 30 µM and up to 12 h after injury [40]. Shelat et al. were able to detect that P188 prevents a variety of detrimental intracellular processes such as activation of caspase activation, mitochondrial cytochrome c release, loss of Δψ_m_, and inhibition of BAX (an effector protein that causes permeabilization of outer mitochondrial membrane) translocation from the cytoplasm to the mitochondria [40]. In another study by Luo et al. 18 h of OGD on mouse primary cortical neurons followed by reoxygenation overnight was chosen as the experimental setup [10]. In different assays, the neurons were treated with P188 at concentrations ranging between 0.1 nM and 100 µM 10 min after OGD [10]. Using a lactate dehydrogenase assay, Luo et al. were able to show that P188 could rescue neurons from OGD-induced cell death starting at a concentration of 10 nM [10]. P188 furthermore inhibited the loss of Δψ_m_ as well as the release of cytochrome c and activation of caspase-3 [10]. These results prove to be highly concordant with Shelat et al.’s, accentuating the potential for direct interaction of P188 with mitochondria [40]. In addition, in a study by Wang et al., embryonic rat hippocampal cells were exposed to 45 min of OGD followed by 2 h of reoxygenation and incubation with fluorescently labeled P188 [18]. Wang et al. were able to observe that P188 localizes to neuronal mitochondria but only in cells subjected to OGD [18].

Because of these previous studies reporting the inhibition of loss of cytochrome c and Δψ_m_, as well as the prevention of BAX translocation from the cytosol to mitochondria, Wang et al.’s study [18] furthermore asked if P188 could inhibit the poration of the outer mitochondrial membrane, specifically. Incubation with 30 nM staurosporine for 24 h was used in mouse embryonic fibroblasts to induce poration of the outer mitochondrial membrane in a cellular model, and incubation with 1.5 nM tBID (a pro-apoptotic protein that induces poration of outer mitochondrial membrane) for 30 min was used to achieve the same in rat isolated brain mitochondria. Fibroblasts or mitochondria were treated with P188 (1, 3, 10, 30, or 100 µM) throughout the 24 h or 30 min, respectively. Mouse embryonic fibroblasts showed increased survival when treated with P188 in a dose-dependent matter with significantly improved survival at a minimum of 10 µM P188 and the highest survival at 100 µm P188. In rat isolated brain mitochondria, cytochrome c release was measured to assess P188′s efficacy. Here, cytochrome c release was significantly reduced in P188-treated mitochondria, starting at a concentration of 10 µM. The strongest reduction in cytochrome c release was seen at 30 µM P188, while 100 µM P188 only reduced cytochrome c release as strongly as 10 µM P188.

Prior to our study, Wang et al.’s study was the only one that directly studied the effects of P188 on isolated mitochondria. However, the positive results of P188 on isolated mitochondria in their study stand in stark contrast to the non-existent effect of P188 shown in the present study. There are, nevertheless, some pronounced differences in the methodical approaches of the two studies. One major distinction is the method of injuring isolated mitochondria: while the ROS H_2_O_2_ used in the present study damages mitochondria at various sites [56], Wang et al. applied tBID to selectively induce poration only of the outer mitochondrial membrane [18]. Accordingly, Wang et al. measured cytochrome c release from mitochondria as this specifically depicts the state of the outer mitochondrial membrane [18]. However, poration of the outer mitochondrial membrane is only one particular component of I/R injury, in which P188 has now proven to be effective [5,18]. In turn, injury of isolated mitochondria with H_2_O_2_ represents a broader spectrum of I/R injury [5,56], but P188 was not able to rescue mitochondrial function in the present study after injuring mitochondria in this way. This poses the question of whether the sole reduction of outer mitochondrial membrane poration seen in Wang et al.’s study [18] can account for the highly improved mitochondrial function after P188 application in vivo, originally seen by Bartos et al. [9]. Another very interesting distinction to be made is the concentration in which P188 was applied: Wang et al. observed the greatest effect at 30 µM P188 and a reduced effect at 100 µM [18]. In conjunction with the results of the present study, this could perhaps suggest that at a concentration of 250 µM or more, P188 may be partly damaging isolated mitochondria, therefore counteracting its own positive properties. While in this specific setting of 200 µM H_2_O_2_ for 10 min, no effects of P188 could be observed, these assumptions could lead the way to further experiments in which P188 is tested on isolated mitochondria at different concentrations and after different injury mechanisms.

### 4.3. Study Limitations

To properly review and discuss the results of the present study, several limitations have to be taken into consideration:

- As shown above, rat isolated brain mitochondria reacted very sensibly to exposure to RT, making this alone a factor that is difficult to account for. The exposure to RT for only 10 more min significantly decreased ATP synthesis of isolated mitochondria. Similarly, in a study by Kleinbongard et al. isolated mitochondria showed lower mitochondrial respiration between a time control sample of mouse isolated heart mitochondria which was exposed to RT for 9 min and a baseline sample which was analyzed directly after isolation [44]. While no further research was found on this specific topic, these results may account for a confounding factor in many studies involving isolated mitochondria, including ours.
- When given after the isolation of mitochondria, P188 did not preserve mitochondrial function after asphyxial CA in vivo. While this concurs with the results achieved in H_2_O_2_-injured mitochondria, it is not known how well the state of mitochondria is being preserved throughout the isolation process and if the state of ischemic damage is actually “frozen” in the mitochondria. Therefore, a possible prolongation of ischemic damage inside the mitochondria could be an explanation for why P188 did not alter mitochondrial function after asphyxial CA in vivo.
- Mitochondrial function was fairly variable. This, generally, poses the question of how much mitochondria become impaired during the isolation process itself. Therefore, it is possible that mitochondria are additionally injured during the isolation such that P188 cannot rescue them anymore.
- As mentioned above, mitochondrial impairment by H_2_O_2_ occurs in a concentration-dependent manner [50]. It may very well be possible that, in the present study, mitochondria were injured too excessively and that a smaller concentration of H_2_O_2_ would have allowed for the injury to be attenuated better by P188.
- In the present study, P188 was only used on mitochondria at concentrations of or exceeding 250 µM. However, in Wang et al.’s study, the optimal concentration seemed to be at 30 µM [18]. Possibly, P188 is only able to attenuate impairment in isolated mitochondria at lower concentrations such as the ones tested in the present study.
- Lastly, the mitochondrial yield from any given animal was typically very low, so that only a few data points per animal could be extracted, making the study resource-intensive and limiting the number of animals used. Thus, insufficient power [57] and a subsequent type II error might explain why some of the results have not achieved significance despite strong trends.

### 4.4. Future Directions

The present study was not able to show a positive effect of P188 at concentrations of 250 or 500 µM on rat isolated brain mitochondria after oxidative stress in vivo or in vitro. From a literature review, however, it becomes apparent that there are many variables to be taken into account when evaluating these results. Thus, future studies should investigate these variables, the first one being the type of injury to the mitochondria.

While isolated mitochondria showed functional impairment after both in vivo and in vitro injury, neither of these injuries was attenuated by P188. As mentioned above, it is not known how well the mitochondrial state is preserved during isolation after ischemic injury; therefore, varying the time span between the end of ischemic injury in vivo and P188 treatment after isolation in vitro could be considered to appraise the extent of this variable. In regard to the in vitro injury, using smaller concentrations of H_2_O_2_ before P188 treatment could clear the question of whether mitochondria were injured too extremely. Furthermore, different injury methods such as anoxia/reoxygenation or Ca^2+^ overload could prove to be more sensitive to P188 treatment [43,44,45,46,48,49,50,51].

After having injured the mitochondria appropriately, the remaining variable is the treatment. For one, Wang et al.’s study displays the importance of finding a concentration that is optimal for P188 [18]. The treatment of mitochondria after simulated I/R injury with different concentrations of P188 is, therefore, of major interest for the future. Furthermore, as the importance of the ratio between the hydrophobic PPO and the hydrophilic PEO within block copolymers becomes more apparent, potential alternatives to P188 are being discovered [16]. For example, diblock copolymers which only have one PEO side-chain attached to the hydrophobic center leave the option to alter the other end of the hydrophobic PPO synthetically, thereby diminishing or increasing its hydrophobicity [16]. This may prove to be more effective than P188 in rescuing cells and perhaps even mitochondria after I/R injury.

## 5. Conclusions

Two methods providing oxidative stress to rat brain mitochondria, namely asphyxial CA in vivo and injury with H_2_O_2_ in vitro, have been used. Contrary to our hypothesis, P188, given upon reoxygenation, did not demonstrate a direct protective effect on mitochondrial function, regardless of the injury method. Due to numerous variables that need to be taken into account, it remains inconclusive whether P188 does or does not directly protect brain mitochondrial function in the context of I/R injury. Further research is needed.

## Data Availability

Data are available from the authors upon request.

## Supplementary Materials

The following are available online at https://www.mdpi.com/2076-3425/11/1/122/s1, Figure S1: Plating Template for Bio-Rad Protein Assay, Figure S2: Strathkelvin Mitocell and oxygen (O_2_) electrode, according to Strathkelvin Instruments Limited [58,59]. Figure S3: CRC assay setup, Table S1: Chemical Substances, Reagents, Solutions, Kits, Table S2: Chemical Ingredients for Isolation Buffer, Table S3: Chemical Ingredients for Experimental Buffer, Table S4: Chemical Ingredients for ATP Assay Buffer.
