# Supplementary material for: Potential Effects of Poloxamer 188 on Rat Isolated Brain Mitochondria after Oxidative Stress In Vivo and In Vitro
Source: Brain Sci. 2021 Jan 18;11(1):122. doi: 10.3390/brainsci11010122 (PMC7831103; doi:10.3390/brainsci11010122)
Supplement: Supplementary file 1 [file brainsci-11-00122-s001.pdf]

brain sciences

Supplementary Materials

Table S1. Chemical Substances, Reagents, Solutions, Kits.

Name	Company	Catalog No.
KetaVed® Ketamine Hydrochloride Injection, USP	Vedco Inc.	50989-996-06
Heparin Sodium Injection, USP	West-Ward Pharmaceuticals	0641-2450-55
D-Mannitol	Sigma-Aldrich	M4125
Sucrose	Sigma-Aldrich	S7903
Potassium phosphate monobasic (KH_2PO_4)	Sigma-Aldrich	P5379
MOPS	Sigma-Aldrich	M3183
Bovine Serum Albumin (BSA)	Fisher Scientific	BP1600
Ethylene glycol-bis(2-aminoethylether)-N,N,N′,N′-tetraacetic acid (EGTA)	Sigma-Aldrich	E4378
Potassium hydroxide (KOH)	Sigma-Aldrich	P1767
Percoll®	Cytiva	17-0891-01
Potassium chloride (KCl)	Sigma-Aldrich	P4504
Potassium phosphate dibasic trihydrate ($K_2HPO_4 \cdot 3H_2O$)	Sigma-Aldrich	P5504
Bio-Rad Protein Assay Dye Reagent Concentrate	Bio-Rad Laboratories, Inc.	5000006
Hydrogen peroxide (H_2O_2) solution	Sigma-Aldrich	216763
Kolliphor® P 188	Sigma-Aldrich	15759
L-Glutamic acid monosodium salt hydrate	Sigma-Aldrich	G1626
L-(−)-Malic acid disodium salt	Sigma-Aldrich	M9138
Sodium succinate dibasic hexahydrate	Sigma-Aldrich	S2378
Rotenone	Sigma-Aldrich	R8875
Dimethyl sulfoxide (DMSO)	Sigma-Aldrich	D5879
P^1,P^5-Di(adenosine-5′) pentaphosphate pentasodium salt (DAP)	Sigma-Aldrich	D4022
Adenosine 5′-diphosphate monopotassium salt dihydrate (ADP)	Sigma-Aldrich	A5285
Adenosine 5′-triphosphate (ATP)	Thermo Fisher	A22066
D-Luciferin sodium salt	Tocris	5427
Luciferase	G-Biosciences	786-1309
Calcium chloride dihydrate	Sigma-Aldrich	C3881
Calcium Green™-5N, Hexapotassium Salt, cell impermeant	Thermo Fisher	C3737

Table S2. Chemical Ingredients for Isolation Buffer (IB).

Concentration	Chemical	Amount per liter
200 mM	Mannitol	36.4 g l^{-1}
50 mM	Sucrose	17.1 g l^{-1}
5 mM	KH_2PO_4	0.68 g l^{-1}
5 mM	MOPS	1.05 g l^{-1}
0.1%	BSA	1 g l^{-1}
1 mM	EGTA	0.38 g l^{-1}

Table S3. Chemical Ingredients for Experimental Buffer (EB).

Concentration	Chemical	Amount per liter
130 mM	KCl	9.7 g l^{-1}
5 mM	$K_2HPO_4 \cdot 3H_2O$[1]	1.14 g l^{-1}
20 mM	MOPS	4.18 g l^{-1}
0.1%	BSA	1 g l^{-1}

[1] For no-phosphate experimental buffer $K_2HPO_4 \cdot 3H_2O$ was excluded.

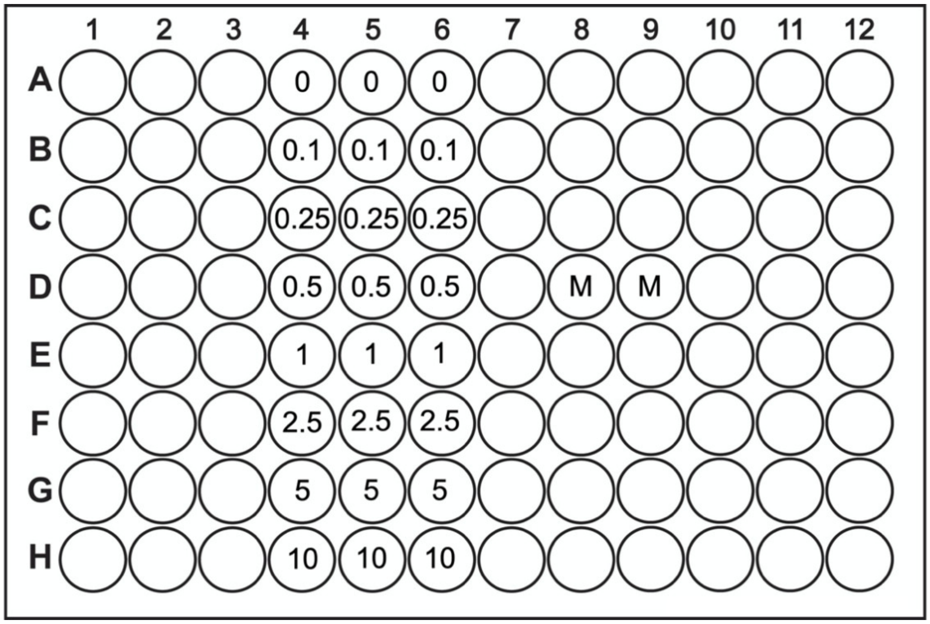

Figure S1. Plating Template for Bio-Rad Protein Assay. The mitochondrial protein concentration was determined by plotting mitochondrial samples (M) against bovine serum albumin (BSA) sample standards of the shown concentrations (in mg ml^{-1}).

Table S4. Chemical Ingredients for ATP Assay Buffer.

Concentration	Chemical
0.2 μM	DAP
30 μM	ADP
0.1 mg ml^{-1}	luciferin
1.25 μg ml^{-1}	luciferase

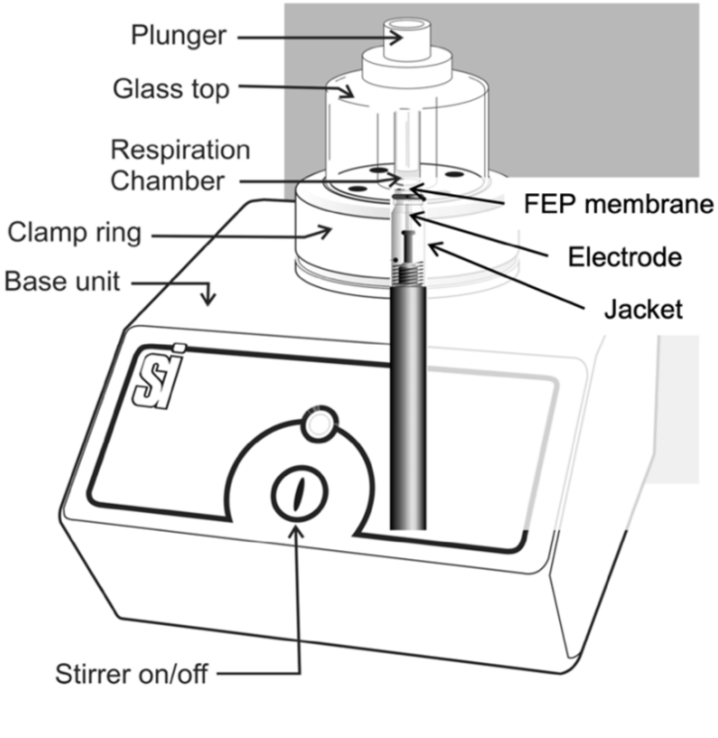

Figure S2. Strathkelvin Mitocell and oxygen (O_2) electrode, according to Strathkelvin Instruments Limited [58,59]. The Mitocell consists of a base unit, which controls the magnetic stirrer and holds the O_2 electrode, and the glass top which forms the respiration chamber and holds a plunger on top. Base unit and glass top are connected through the clamp ring. The electrode swims in a buffered potassium chloride (KCl) solution and is covered by a jacket. The electrode is separated from the respiration chamber by the fluorethylenpropylen (FEP) membrane, which is highly but selectively permeable for O_2. Mitochondrial O_2 consumption over time is therefore measurable and the mitochondrial viability determinable.

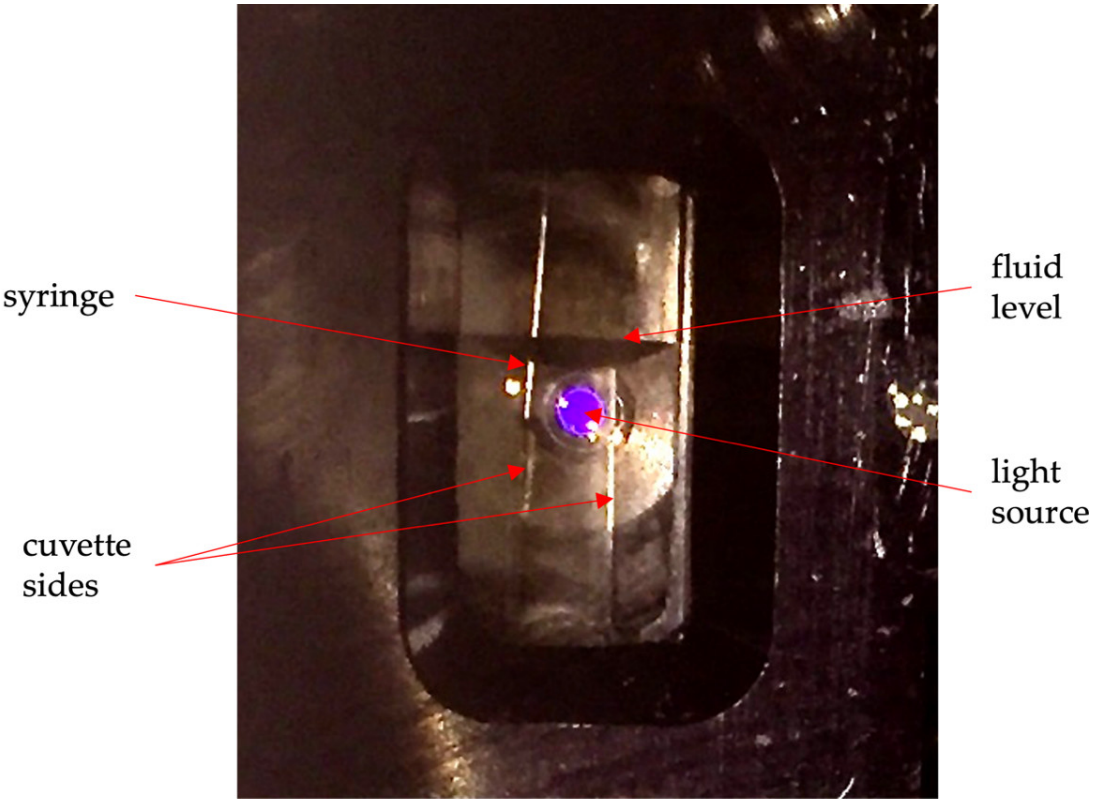

Figure S3. Calcium retention capacity (CRC) assay setup. The needle infusing the calcium chloride ($CaCl_2$) had to be inserted deep enough into the cuvette so that the $CaCl_2$ would not remain on top of the fluid level but instead be well mixed with the fluid in the cuvette. However, the needle was not allowed to cross the light path; therefore, the needle was inserted at a slight angle to rest at the side of the cuvette.
